# Effect of combined Kinesiotaping and resistive exercise on muscle strength and quality of life in breast cancer survivors: a randomized clinical trial

**DOI:** 10.1186/s43046-023-00205-z

**Published:** 2024-01-15

**Authors:** Alaa M. Ramadan, Abeer M. ElDeeb, Ahmed A. Ramadan, Dina M. Aleshmawy

**Affiliations:** 1 Department of Physical Therapy for Obstetrics and Gynecology, Faculty of Physical Therapy, October 6 University, Giza, Egypt; 2 Department of Physical Therapy for Women’s Health, Faculty of Physical Therapy, Cairo University, Giza, Egypt; 3 Department of Surgery, Faculty of Medicine, Suez Canal University, Ismailia, Egypt

**Keywords:** Breast cancer, Chemotherapy, Kinesiotaping, Resistive exercise, Muscle strength, Quality of life

## Abstract

**Background:**

Breast cancer (BC) and its treatment affect women's tissue architecture and physiology, which leads to impaired muscle strength and joint dysfunction, affecting quality of life (QOL). Most evidence has focused on exercises; however, due to the complexity and heterogeneity of patients’ rehabilitation needs, further research is required to investigate more adjunctive methods to help optimal rehabilitation according to patients’ needs, preferences, and effective interventions.

**Methods:**

This study aimed to determine the effect of Kinesiotaping (KT) combined with resistive exercise on muscle strength and QOL in breast cancer survivors (BCS). Forty premenopausal BCS treated with chemotherapy postmastectomy participated in this study. Their age ranged from 40 to 55 years, and their body mass index (BMI) was 25–29.9 kg/m^2^. They were randomly distributed into two equal groups. The control group received resistive exercise two times/week for 12 weeks, while the study group received resistive exercise and KT applied to the lower limbs. Hip, knee, and ankle muscle strength were measured using a hand-held dynamometer, and QOL was evaluated using 36-Item Short Form (SF-36) before and after treatment.

**Results:**

Both groups showed a significant increase (*p* = 0.0001) in the strength of hip flexors, knee extensors, flexors, ankle plantar flexors, and dorsiflexors, as well as SF-36 score after treatment. However, the study group showed a more significant increase in strength of hip flexors (*p* = 0.005), knee extensors (*p* = 0.01) and flexors (*p* = 0.02), ankle plantar flexors (*p* = 0.01), and dorsiflexors (*p* = 0.01), as well as SF-36 score (*p* = 0.006) than the control group.

**Conclusions:**

KT plus resistive exercise is more effective than exercise alone for improving muscle strength and QOL in BCS. So, the KT can be recommended as a non-invasive, adjunctive method added to the protocol therapy for BCS to help better outcomes during the rehabilitation period.

## Background

Breast cancer (BC) is the most prevalent cancer in women all over the world. Advancements in diagnostics and treatment have led to a notable rise in the survival rate, which presents novel challenges for healthcare providers to help patients attain optimal rehabilitation [1]. Several factors increase the risk of developing cancers, which include a sedentary lifestyle, smoking, alcoholism [2], or obesity [3].

Most patients suffer from a muscle-wasting syndrome called cachexia [4] before and after cancer treatment [5]. Cachexia is the continued loss of muscle mass with or without weight loss [6]; it results from systemic inflammation and catabolic stimuli, inhibiting protein synthesis and enhancing muscle catabolism [7].

Additionally, the use of chemotherapy may result in decreased muscle strength and joint dysfunction, which impact the quality of life (QOL). Patients showed 25% lower strength in the maximal voluntary isometric contraction of lower extremities compared with healthy women [8] and reported a reduction of QOL in the aspects of physical function, and body image [9]. Decreased muscle strength and mass negatively affect patients’ performance status [8], tolerance to chemotherapy, and prognosis in both medical and surgical cancer patients [10], and lead to morbidity and mortality in advanced cancer [4, 6, 8].

Most of the research has focused on how exercise can help cancer patients manage chemo-related adverse effects [11] such as fatigue, anxiety, poor QOL [12], and impaired muscle strength. Despite the benefits of resistive exercises, and due to the complexity of the patient’s rehabilitation needs, further clinical studies are required to individualize treatment [13] and to determine the best rehabilitation for patients based on their needs, preferences, and effective interventions [14, 15]. Furthermore, a recent systematic review found that maximum benefits in muscle strength and hypertrophy require light loads with high repetitions or high-load training, which might not be suitable for all subjects. It suggested that other approaches for maximizing motor unit recruitment may be just as productive without performing high repetitions with light loads [16].

Kinesiotaping (KT) is a therapeutic modality used alone or combined with exercise, which improves isokinetic muscle strength due to its facilitative action on the muscles [17, 18]. KT showed superiority over other non-invasive interventions for muscle fatigue recovery [19], increased isometric muscle strength, and decreased muscle soreness after intensive exercise compared with the placebo and stretch groups [20]. However, there is a need for more high-quality and long intervention period research describing the application direction and the tension amount used [17, 18].

To the best of our knowledge, there were no studies investigating the effect of adding KT to strength exercises in BCS to determine the best clinical intervention, that would counteract and improve chemotherapy-related side effects and improve functional performance during the rehabilitation period. Therefore, the present study aimed to investigate the effect of combined KT and resistive exercise on muscle strength and QOL in BCS compared to resistive exercise alone. The hypothesis was that adding KT to resistive exercise would improve muscle strength and QOL than exercise alone in BCS.

## Methods

### Participants

The study is a prospective, controlled, randomized clinical trial. A physician recruited a sample of 46 sedentary overweight middle-aged women diagnosed with BC stage I–III from the Outpatient Clinic, Oncology Hospital. Their ages ranged from 40 to 55 years, and their body mass index (BMI) was 25 to 29.9 kg/m^2^. They had regular menstrual cycles. All BCS received chemotherapy for 1–12 weeks after mastectomy. The exclusion criteria included respiratory or heart problems affecting mobility, lymphedema, marked skeletal deformity, neuropathy, visual system affection, cognition problems, previous surgeries at their back or lower limbs, or skin over-sensitivity to tape.

**Fig. 1:**
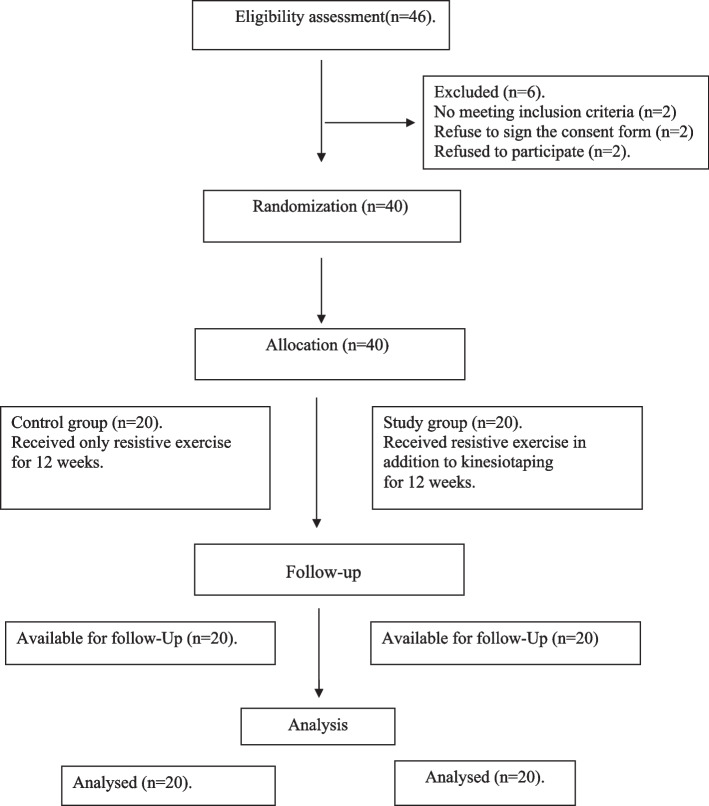
Flow diagram chart

Out of 46 participants, two did not meet the inclusion criteria, two refused to sign a consent form, and two did not accept to join the study (Fig. 1). A blinded investigator created a computer-generated simple randomization sequence to allocate participants using a sealed envelope procedure to the control or study group. The control group performed a resistive exercise program two times/week for 12 weeks, while the study group received the same program and application of KT for 12 weeks. However, due to the nature of the intervention, the participants were aware of the group allocation. The data analysis and the primary outcome assessors were blinded to the participant allocation. No subjects dropped out of the study.

Based on a prior study [21], the sample size was calculated using the statistical program G*POWER (version 3.1.9.2; Franz Faul, University Kiel, Germany). It revealed that the sample size required was 20 subjects in each group with α = 0.05, power = 80%, and effect size = 0.91.

### Outcome measures

#### 1-Assessment of muscle strength

A hand-held dynamometer (HHD) (Lafayette Instrument Company, IN 47904 USA) was the method used to record the peak isometric force of the hip flexors, knee flexors, and extensors, as well as ankle plantar flexors and dorsiflexor muscles. It consisted of a lightweight (10.6 oz) microprocessor-control unit that measured peak force in kilograms while storing up to 52 tests. It allowed test times ranging from 1–10 s and had an audible tone indicating the end of the preset time. The unit provided a built-in calibration routine that verified a valid calibration. It was a valid and reliable tool for measuring lower limb muscle isometric strength among adults [22, 23].

Testing positions and strength assessments were performed as described by Mentiplay et al. [24]. Before starting, the manufacturer validated the dynamometer. Each patient maintained contraction for each muscle group for 5 s with a one-minute rest between measurements to decrease fatigue impact. An examiner blinded to the patient’s assignment performed the measurements three times and recorded the averages before and after 12 weeks of the treatment.

#### 2-Assessment of QOL

The most common tool used to measure health-related QOL, especially among cancer patients, is the 36-item short-form survey (SF-36). This survey evaluated perceived health status across broad physical and emotional health domains. It comprised 36 questions that covered eight domains of health in BCS. It assessed physical functioning, role limitations due to physical health, bodily pain, general health, energy/fatigue, social functioning, role limitations due to emotional problems, and emotional well-being. Each patient received thorough instructions about the survey and enough time to fill it out. She gave each question a score between 0 and 100, with 100 indicating the maximum degree of functioning. The investigator calculated the final score by averaging the results of the questions that addressed each domain [25] before and after the 12-week course of treatment.

### Interventions

#### A-Resistive exercises

The resistive exercise program included warm-up, active, and cool-down phases. Warm-up and cool-down phases consisted of 5 to 10 min of stretching exercise for the pectorals major, hamstring, hip flexors, and calf muscles, with each static stretch lasting at least 20 s in the first week and then 30 s for the remaining 11 weeks. In the active phase, strengthening exercises using sandbags were applied for hip flexors, knee flexors and extensors, and ankle plantar flexors and dorsiflexors. The therapist determined one repetition maximum (1RM) for each muscle group, which was the maximum weight the patient lifted for one time. Then, the load applied was 50–80% of 1RM along the treatment course. Each exercise repetition was 8–12 times, and the number of sets (1–3) continuously increased over the first 2–3 weeks. The program lasted approximately 60 min, twice a week, for 12 weeks [13].

#### B-Kinesiotaping application

Elastic Ares KT (made in Korea) was applied to the muscles of the lower extremities. For sartorius, the I-strip extended from the anterior superior iliac spine to the medial edge of the patella; for rectus femoris, the I-strip extended from the anterior inferior iliac spine to the patella upper edge [26]. Also, two I-strip were applied, one for the semimembranosus/semitendinosus from the ischial tuberosity to the posterior surface of the medial tibial condyle and the other for the biceps femoris from the ischial tuberosity to the fibular head posterior surface [27]. The patellar KT application used two Y strips cut about 2/3 of the length of the strip down the middle to create two tails; the uncut portion of one Y-strip was above the superior portion of the patella with the tails winged medially and laterally to the medial and lateral femoral condyles, respectively; the other uncut portion was above on the tibial tuberosity with the tails winged medially and laterally around the patella [28]. The tibialis anterior KT application used one I-strip extending from the tibia proximal lateral portion to the first metatarsal and medial cuneiform [29]. Each strip was placed with a 20–25% stretch and downward pressure, with no tension applied to the first and last inch of the strip. The study group received KT applications two times/week for 12 weeks.

### Statistical analysis

The Statistical Package for Social Studies (SPSS) version 25 for Windows (IBM SPSS, Chicago, IL, USA) was the software used for all statistical analysis. The Shapiro–Wilk test evaluated the normal distribution of data. Levene’s test for homogeneity of variances revealed the homogeneity between groups. Mixed MANOVA was the method used to investigate the effect of treatment. The Bonferroni correction was the post-hoc test used for subsequent multiple comparisons. The level of significance for all statistical tests was at *p* < 0.05.

## Results

Table 1 represents the patient characteristics of the control and study groups; unpaired *t*-tests showed no significant difference between groups in age (*p* = 0.16), weight (*p* = 0.16), height (*p* = 0.24), BMI (*p* = 0.29), and duration of chemotherapy (*p* = 0.46).

**Table 1:** Comparison of patients’ characteristics between the control and study groups

Variables	Control group (*N* = 20)	Study group (*N* = 20)	MD	*t* value	*p* value
Age (years)	51.05 ± 4.27	48.95 ± 5.05	2.10	1.42	0.16^NS^
Weight (kg)	74.03 ± 6.97	77.13 ± 6.82	− 3.10	− 1.42	0.16^NS^
Height (cm)	164 ± 6.41	162 ± 4.95	2	1.19	0.24^NS^
BMI (kg/m^2^)	27.4 ± 1.34	28.22 ± 2.77	− 0.75	− 1.06	0.29^NS^
Duration of chemotherapy (weeks)	5.73 ± 4.25	6.75 ± 4.48	− 1.03	− 0.74	0.46^NS^

*MD* mean difference, *p* value probability value, *NS* non-significant, *BMI* body mass index

As shown in Table 2, the control and study groups showed a significant increase (*p* = 0.001) in the strength of hip flexors, knee extensors and flexors, ankle plantar flexors, and dorsiflexors, as well as the SF-36 score after treatment. Compared to the control group, the study group showed a significant increase in the strength of hip flexors (*p* = 0.005), knee extensors (*p* = 0.01), flexors (*p* = 0.02), ankle plantar flexors, and dorsiflexors (*p* = 0.01), as well as the SF36 score (*p* = 0.006). None of the patients reported discomfort, limitations of motion, or other side effects while using KT.

**Table 2:** Mean values of the muscle strength and SF36 score of the control and study groups before and after treatment

Variables		Before	After	MD	% of change	*p* value
Hip flexors strength	Control group	7.06 ± 0.69	7.85 ± 0.71	− 0.79	11.19	0.001^*^
	Study group	7.03 ± 0.66	8.46 ± 0.57	− 1.43	20.34	0.001^*^
	MD	0.03	− 0.61			
	*p* value	*p* = *0.87*^*NS*^	*p* = 0.005^*^			
Knee extensors strength	Control group	5.08 ± 0.80	5.70 ± 0.93	− 0.62	12.20	0.001^*^
	Study group	5.00 ± 0.73	6.45 ± 0.93	− 1.45	29	0.001^*^
	MD	0.08	− 0.75			
	*p* value	*p* = *0.74*^*NS*^	*p* = 0.01^*^			
Knee flexors strength	Control group	4.35 ± 0.48	4.81 ± 0.60	− 0.46	10.57	0.001^*^
	Study group	4.50 ± 0.58	5.32 ± 0.79	− 0.82	18.24	0.001^*^
	MD	− 0.14	− 0.50			
	*p* value	*p* = *0.39*^*NS*^	*p* = 0.02^*^			
Ankle planter flexors strength	Control group	3.67 ± 0.62	4.07 ± 0.70	− 0.40	10.9	0.001^*^
	Study group	3.55 ± 0.58	4.68 ± 0.75	− 1.14	31.83	0.001^*^
	MD	0.12	− 0.61			
	*p* value	*p* = *0.51*^*NS*^	*p* = 0.01^*^			
Ankle dorsiflexors strength	Control group	3.33 ± 0.81	3.76 ± 0.77	− 0.43	12.91	0.001^*^
	Study group	3.36 ± 0.72	4.35 ± 0.72	− 0.99	29.46	0.001^*^
	MD	− 0.03	− 0.59			
	*p* value	*p* = *0.88*^*NS*^	*p* = 0.01^*^			
SF-36 score	Control group	44.82 ± 8.13	69.89 ± 8.85	− 25.07	55.95	0.001^*^
	Study group	42.45 ± 8.13	77.50 ± 7.73	− 35.05	82.56	0.001^*^
	MD	2.37	− 7.61			
	*p* value	*p* = *0.36*^*NS*^	*p* = 0.006^*^			

*MD* mean difference, *p* value probability value, *NS* non-significant, *SF-36* Short form 36

^*^Significant at *p* < 0.05

## Discussion

BC women suffer from many adverse effects resulting from chemotherapy such as fatigue, and impaired muscle strength, which affect QOL [30]. Due to complicated patient rehabilitation needs, further studies are warranted to investigate adjunctive modalities to achieve maximum benefits for patients’ outcomes during the treatment course. Therefore, the current study aimed to determine the effect of adding KT to resistive exercise on the lower limb muscle strength and QOL compared to exercise alone in BCS. Results revealed that adding KT to resistive exercise showed more improvement in the hip flexors, knee flexors, extensors, ankle plantar flexors, and dorsi flexors strengths, as well as QOL than resistive exercise alone.

A recent systematic review [11] confirmed the results about the efficacy of resistive activities. It suggested resistive exercise as part of supportive treatment for BCS following anticancer treatment because it improved QOL, muscle strength, endurance, physical performance, mental function, and leisure time in BCS. Exercise-induced benefits could be related to lowered cytokines, which play a significant role in cancer development; however, this was not addressed in this study [31]. Resistive exercise may counteract some mechanisms underlying muscle wasting in cancer patients by reducing the production of pro-inflammatory cytokines and enhancing the phosphorylation of intramuscular amino acid-signaling molecules [32, 33]. Also, exercise enhances metabolism and metabolic phenotype in a variety of tissues, which are mediated in part by transcriptional responses [34]; the increase in PGC1α, a transcriptional coactivator, in response to exercise, activates branched-chain amino acid (BCAA) metabolism, which increases protein synthesis [35]. Increased muscle content of proteins is associated with enhanced protein stability and may be attributed to a chaperone-dependent mechanism and/or reduced regulation by proteolysis [36]. These adaptive responses increase oxidative capacity [34]. Moreover, resistive exercise increases the activity of cellular antioxidant enzymes such as superoxide dismutase 1 (SOD1), SOD2, catalase (CAT), and peroxidase (GPX), which improve antioxidant capacity and protect against oxidative stress induced by contraction, delaying muscle fatigue [37]. Moreover, increased muscle strength may result from neuromuscular adaptations resulting from increased motoneuron recruitment and excitability which enhance muscle cell activation during exercises [16].

KT plus resistive exercises improved QOL, which was a significant predictor of better treatment outcomes [38]. Previous research showed the same finding; one study has reported that KT applied for 5 weeks achieved significant change in QOL variables, which included global health, fatigue, pain, and well-being in BCS treated with an Aromatase inhibitor [39]. Another study has shown that KT and lifestyle changes improved physical function, pain, and QOL in women with dysmenorrhea [40].

Regarding the effect of KT on muscle strength, the results confirmed earlier findings of previous studies. They reported that KT increased isometric muscle strength and reduced muscle soreness after intensive exercise [20], and increased muscle torque of the vastus medialis, laterals, and rectus femoris [26].

However, these findings contraindicated previous studies; they found no substantial difference in QOL [41] in women with chronic venous insufficiency and the quadriceps muscle strength after short application of KT in healthy subjects [19] and patients with osteoarthritis [17, 18]. These contradictions may result from the difference in the participants’ muscle strength and duration of the KT treatment among studies.

The more significant increase in the muscle strength in the group receiving KT and exercise than exercise alone may result from the additive facilitative action of KT on the muscles, which was supported by the electromyography (EMG) findings of increased activity of the vastus medialis muscle following 24 h of using KT [26]; KT could induce tactile stimulation, which results in the firing of large-diameter afferent fibers and the inhibition of small-diameter afferent fibers, hence limiting pain transmission, decreasing muscular soreness, and improving muscle strength. It may also help with muscle strengthening by transmitting a pulling force to the muscle and fascia and activating mechanoreceptors; application of KT in the direction of the muscle contraction could enhance the muscle spindle reflex and increase the excitability of the motor units, improving the intensity of the muscle stimulation. Also, KT could raise the skin away from the underlying fascia, promoting blood and lymphatic flow, which enhances muscle activity and increases oxygen distribution to the muscle. Also, KT could improve muscle fatigue recovery after exercise, enhancing exercise performance [19]. However, the physiological underlying mechanisms, such as protein synthesis and antioxidant enzyme activity in response to KT application, are still unknown and require more research.

## Limitations

There are some limitations to the current study. Firstly, this study does not investigate the mechanisms underlying the effect of combined resistive exercise and KT. Therefore, further research is warranted to investigate the effect of combined interventions on cytokines in BCS. Secondly, the study is restricted to a narrow range of women’s ages and BMI, so more research is needed to assess the effect of the interventions using large sample sizes with different ages, a wide range of BMI, and more objective instruments like EMG to investigate muscle activity and isokinetic to assess muscle strength and torque. Thirdly, the long-term effect of the interventions and follow-up in BCS needs to be investigated. Fourthly, there is a need to determine the effect of the application of KT to the back muscles on back muscle activities, strength, and torque in BCS.

## Conclusions

KT plus resistive exercise is more effective than exercise alone to counteract and improve related side effects of chemotherapy such as impaired muscle strength and poor QOL. KT can be added to the protocol therapy of cancer as it enhances the functional performance of the BCS.

## Data Availability

The data that support the findings of this study are available from the corresponding author upon reasonable request.

## Abbreviations

BC: Breast cancer
BCS: Breast cancer survivors
BMI: Body mass index
HHD: Hand-held dynamometer
KT: Kinesiotaping
1RM: One repetition maximum
QOL: Quality of life
